# Crystal structure, DFT and MEP study of (*E*)-2-[(2-hy­droxy-5-meth­oxy­benzyl­idene)amino]­benzo­nitrile

**DOI:** 10.1107/S2056989019008077

**Published:** 2019-06-14

**Authors:** Md. Serajul Haque Faizi, Necmi Dege, Ceren Çiçek, Erbil Agar, Igor O. Fritsky

**Affiliations:** a PG Department of Chemistry, Langat Singh College, B. R. A. Bihar University, Muzaffarpur, Bihar-842001, India; b Ondokuz Mayıs University, Faculty of Arts and Sciences, Department of Physics, 55139, Kurupelit, Samsun, Turkey; c Ondokuz Mayıs University, Faculty of Arts and Sciences, Department of Chemistry, 55139, Kurupelit, Samsun, Turkey; d National Taras Shevchenko University, Department of Chemistry, Volodymyrska str., 64, 01601 Kyiv, Ukraine

**Keywords:** crystal structure, 2-hy­droxy-5-meth­oxy­benzaldehyde, weak hydrogen bonding, 2-amino­benzo­nitrile

## Abstract

The asymmetric unit of the title compound, C_15_H_12_N_2_O_2_, contains two crystallographically independent mol­ecules in which the dihedral angles between the benzene rings in each are 13.26 (5) and 7.87 (5)°. An intra­molecular O—H⋯N hydrogen bonds results in the formation of an *S*(6) ring motif. In the crystal, mol­ecules are linked by weak C—H⋯O and C—H⋯N hydrogen bonds, forming layers parallel to (011). In addition, π–π stacking inter­actions with centroid–centroid distances in the range 3.693 (2)–3.931 (2) Å complete the three-dimensional network.

## Abstract 2

The asymmetric unit contains two crystallographically independent mol­ecules in which the dihedral angles between the benzene rings are 13.26 (5) and 7.87 (5)°. An intra­molecular O—H⋯N hydrogen bonds results in the formation of an *S*(6) ring motif. In the crystal, mol­ecules are linked by weak C—H⋯O and C—H⋯N hydrogen bonds, forming layers parallel to (011). π–π stacking inter­actions complete the three-dimensional network.

## Chemical context   

Most Schiff bases have anti­bacterial, anti­cancer, anti inflammatory and anti­toxic properties (Williams, 1972 ▸). In addition, Schiff bases are important in diverse fields of chemistry and biochemistry owing to their biological activities (Lozier *et al.*, 1975 ▸). On the industrial scale, they have a wide range of applications, such as in dyes and pigments, and Schiff bases have also been employed as ligands for the complexation of metal ions (Taggi *et al.*, 2002 ▸). Photochromism and thermochromism are also characteristics of these materials and arise *via* H-atom transfer from the hy­droxy O atom to the N atom (Hadjoudis *et al.*, 1987 ▸). In NLO studies, Schiff base provide the key functions of frequency shifting, optical modulation, optical switching, optical logic, and optical memory for the emerging technologies in areas such as telecommunications, signal processing, and optical inter­connections (Geskin *et al.*, 2003 ▸). The present work is a part of an ongoing structural study of Schiff bases and their utilization in the synthesis of quinoxaline derivatives (Faizi *et al.*, 2016*a* ▸), fluorescence sensors (Faizi *et al.*, 2016*b* ▸) and coordination compounds (Faizi & Prisyazhnaya, 2015 ▸).
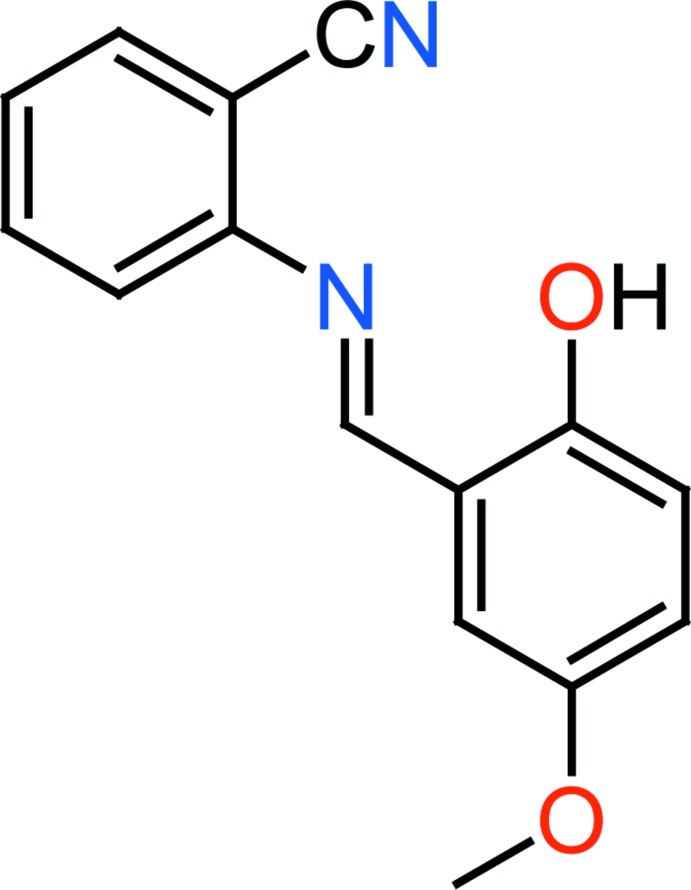


We report herein on the synthesis, crystal structure and DFT computational calculation of the new title Schiff base compound, (I). The results of calculations by density functional theory (DFT) on (I) carried out at the B3LYP/6–311G(d,p) level are compared with the experimentally determined mol­ecular structure in the solid state.

## Structural commentary   

**Figure 1:**
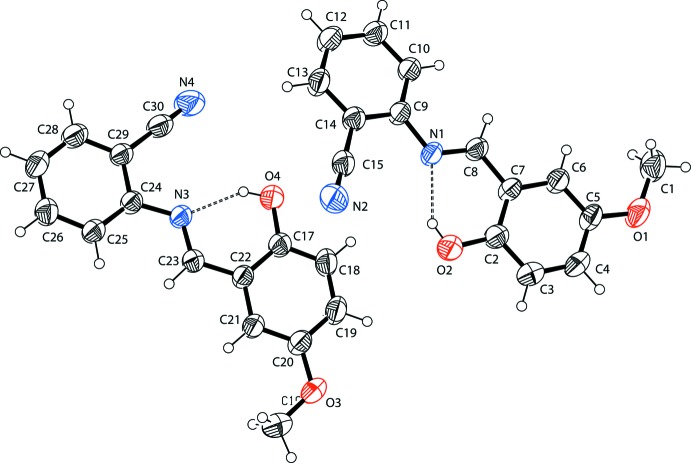
The mol­ecular structure of the title mol­ecule, with the atom-numbering scheme. Displacement ellipsoids are drawn at the 40% probability level. The intra­molecular O—H⋯N hydrogen bonds (Table 1 ▸) are shown as dashed lines.

The asymmetric unit of the title compound contains two crystallographically independent mol­ecules (Fig. 1 ▸; r.m.s. deviation of overlay of the two molecules = 0.035 Å) in which the bond lengths (Allen *et al.*, 1987 ▸) and angles are normal and in good agreement with those reported for 5-chloro-2-(2-hy­droxy­benzyl­idene­amino)­benzo­nitrile (Cheng *et al.*, 2006 ▸) and 2-(2-hy­droxy­benzyl­idene­amino) benzo­nitrile (Xia *et al.*, 2008 ▸). The benzene rings in the two independent mol­ecules [*A* (C2–C7)/*B* (C9–C14) and *C* (C17–C22)/*D* (C24–C29)] subtend dihedral angles *A*/*B* = 13.26 (5) and *C*/*D* = 7.87 (5)°. The title compound displays a *trans* configuration with respect to the C8=N1 and C23=N3 double bonds. In each independent mol­ecule, an intra­molecular O—H⋯N hydrogen bond (Table 1 ▸) results in the formation of a planar six-membered ring [*G* (N1/H2/O2/C2/C7/C8) and *H* (O4/H4/N3/C23/C22/C17)]; these are oriented at dihedral angles of *A*/*G* = 1.31 (5) and *C*/*H* = 0.42 (5)° with respect to the adjacent benzene rings.

## Supra­molecular features   

**Figure 2:**
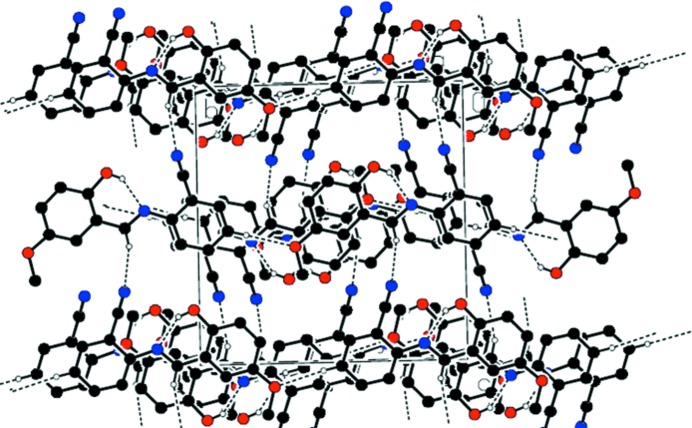
Part of the crystal structure with weak C—H⋯O and C—H⋯N hydrogen bonds shown as dashed lines.

**Figure 3:**
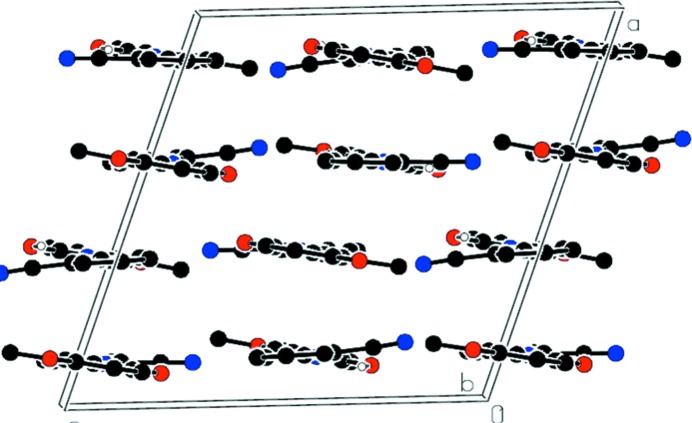
Part of the crystal structure viewed along the *b* axis to illustrate the π–π stacking inter­actions in the crystal. label for c axis not visible

In the crystal, weak C—H⋯O hydrogen bonds link both types of independent mol­ecule into chains along [010] while weak C—H⋯N hydrogen bonds link the chains into a two-dimensional network parallel to (011) (Fig. 2 ▸ and Table 1 ▸). In addition, three types of *π–π* stacking inter­actions occur between benzene rings: *Cg*1⋯*Cg*3(−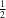 + *x*, 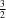 − *y*, −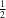 + *z*) = 3.860 (2) Å, *Cg*2⋯*Cg*2(1 − *x*, 1 − *y*, −*z*) = 3.693 (2) Å and *Cg*2⋯*Cg*4(−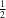 + *x*, 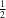 − *y*, −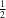 + *z*) = 3.931 (2) Å; where *Cg*1, *Cg*2, *Cg*3 and *Cg*4 are the centroids of the C2–C7, C9–C14, C17–C22 and C24–C29 rings, respectively (Fig. 3 ▸).

## Frontier mol­ecular orbital analysis   

**Figure 4:**
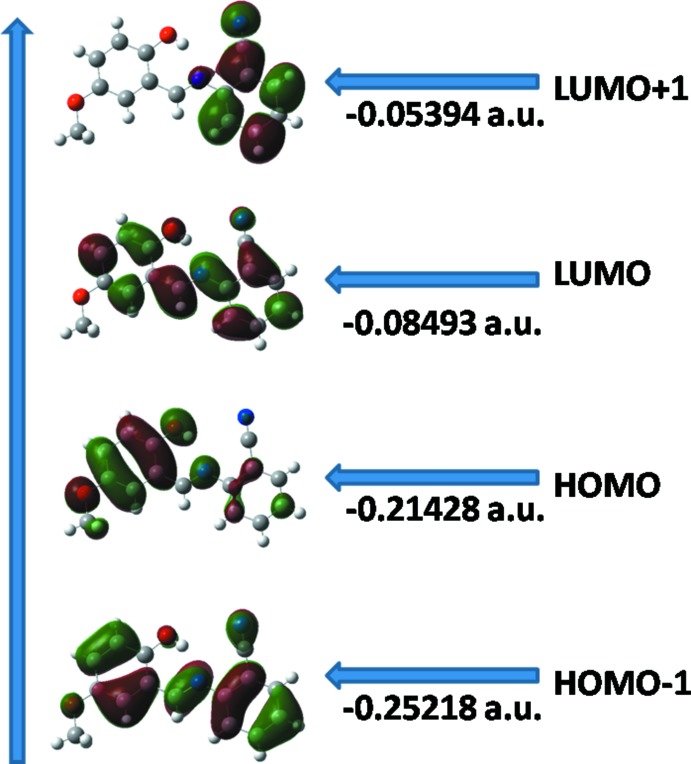
Mol­ecular orbital surfaces and energies of HOMO−1, HOMO, LUMO and LUMO+1 for (I).

The highest occupied mol­ecular orbitals (HOMOs) and the lowest lying unoccupied mol­ecular orbitals (LUMOs) are termed frontier mol­ecular orbitals (FMOs), which play an important role in the optical and electric properties of compounds, as well as in their quantum chemistry and UV–vis spectra. According to mol­ecular orbital theory, an inter­action between HOMO and LUMO orbitals of a structure gives rise to a π–π* type transition. The frontier orbital gap helps to characterize the chemical reactivity and the kinetic stability of the mol­ecule. A mol­ecule with a small frontier orbital gap is generally associated with a high chemical reactivity, low kinetic stability and is also termed a soft mol­ecule. DFT quantum-chemical calculations for the title compound were performed at the B3LYP/6–311G(d,p) level (Becke, 1993 ▸) as implemented in *GAUSSIAN09* (Frisch *et al.*, 2009 ▸). The DFT structure optimization started from the X-ray geometry and the experimental bond lengths and bond angles were found to match with theoretical values indicating that the 6-311G(d,p) basis set is well suited in its approach to the experimental data. The DFT study of (I) shows that the HOMO and LUMO are localized in the plane extending from the whole phenol ring to the cyano benzene ring. The electron distribution of the HOMO−1, HOMO, LUMO and the LUMO+1 energy levels are shown in Fig. 4 ▸. The HOMO mol­ecular orbital exhibits both σ and π character, whereas HOMO−1 is dominated by π-orbital density. The LUMO is mainly composed of π density while LUMO+1 has both σ and π electronic density. The HOMO–LUMO gap is 0.12935 a.u. and the frontier mol­ecular orbital energies, *E*_HOMO_ and *E*_LUMO_ are −0.21428 and −0.08493 a.u., respectively.

## Mol­ecular electrostatic potential surface analysis   

**Figure 5:**
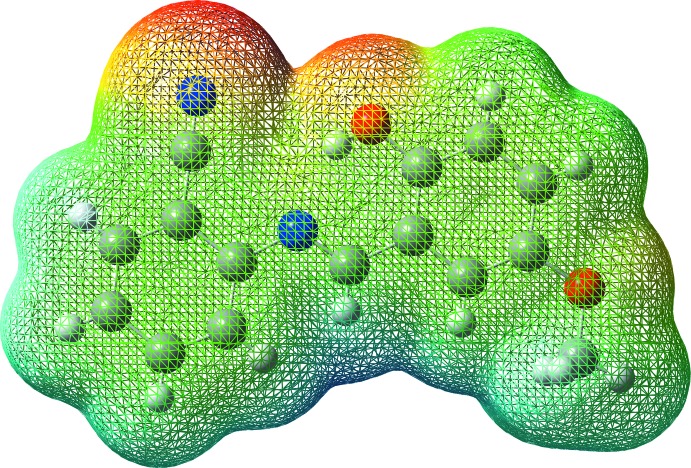
Total electron density mapped over the mol­ecular electrostatic potential surface.

Mol­ecular electrostatic potential (MEP) surface analysis is a technique of mapping electrostatic potential onto the iso-electron density surface, providing information about the reactive sites. The surface simultaneously displays mol­ecular size and shape and the electrostatic potential value. In the colour scheme adopted, red indicates an electron-rich region with a partially negative charge and blue an electron-deficient region with partially positive charge, light blue indicates a slightly electron-deficient region, yellow a slightly electron-rich region and green a neutral region (Politzer *et al.*, 2002 ▸). In addition to these, in the majority of the MEPs, the maximum positive region, which is the preferred site for nucleophilic attack, is shown in blue and the maximum negative region, which is preferred site for electrophilic attack, is red. A three-dimensional plot of the MEP surface of one of the two independent molecules of the title compound is shown in Fig. 5 ▸. According to this, the negative regions of the mol­ecule are located on the donor oxygen atom, the acceptor nitro­gen atom and the benzo­nitrile group of N2 atom (red region). The positive regions over the meth­oxy hydrogen atoms and all other hydrogen atoms indicate that these sites are most probably involved in nucleophilic processes.

## Database survey   

A search of the Cambridge Structural Database (CSD, version 5.39; Groom *et al.*, 2016 ▸) gave eight hits for the (*E*)-2-[(2-hy­droxy-5-meth­oxy­benzyl­idene)amino]­benzo­nitrile moiety: (*Z*)-2-[(2-hy­droxy-1-naphth­yl)methyl­ene­amino]­benzo­nitrile (FOVRUE; Zhou *et al.*, 2009*c* ▸), (*E*)-2-[(5-bromo-2-hy­droxy­benzyl­idene)amino]­benzo­nitrile (FOWXOF; Zhou *et al.*, 2009*b* ▸), 5-chloro-2-(2-hy­droxy­benzyl­idene­amino)­benzo­nitrile (GEJGAE; Cheng *et al.*, 2006 ▸), *trans*-2-(2-hy­droxy­benzyl­id­ene­amino)­benzo­nitrile­(LOCBOV; Xia *et al.*, 2008 ▸), 2-[(2-hy­droxy-6-meth­oxy­benzyl­idene)amino]­benzo­nitrile (LOVDUX; Demircioğlu *et al.*, 2015 ▸), (*E*)-2-(2,4-di­hydroxy­benzyl­idene­amino)­benzo­nitrile (MOZPAT; Liu 2009 ▸), (*E*)-2-(4-di­ethyl­amino-2-hy­droxy­benzyl­idene­amino)­benzo­nitrile (PUJDOO; Wang *et al.*, 2010 ▸) and (*E*)-2-[(3,5-di-*tert*-butyl-2-hy­droxy­benzyl­idene)amino]­benzo­nitrile (YOVBUH; Zhou *et al.*, 2009*a* ▸). In all of these compounds, an intra­molecular O—H⋯N hydrogen bond forms an *S*(6) ring motif, similar to title compound.

## Synthesis and crystallization   

The title compound was prepared by refluxing mixed solutions of 2-hy­droxy-5-meth­oxy­benzaldehyde (38.0 mg, 0.25 mmol) in ethanol (15 ml) and 2-amino­benzo­nitrile (29.5 mg, 0.25 mmol) in ethanol (15 ml). The reaction mixture was stirred for 5 h under reflux. Single crystals of the title compound suitable for X-ray analysis were obtained by slow evaporation of an ethanol solution (yield 60%, m.p. 414–416 K).

## Refinement   

Crystal data, data collection and structure refinement details are summarized in Table 2 ▸). H atoms were positioned geometrically (O—H = 0.82, C—H = 0.93–0.96 Å) and refined as riding with *U*_iso_(H) = 1.2*U*_eq_(C) or 1.5*U*_eq_(O,C-meth­yl).

## Supplementary Material

Crystal structure: contains datablock(s) I. DOI: 10.1107/S2056989019008077/lh5907sup1.cif

Structure factors: contains datablock(s) I. DOI: 10.1107/S2056989019008077/lh5907Isup2.hkl

Supporting information file. DOI: 10.1107/S2056989019008077/lh5907Isup3.cml

CCDC reference: 1912294

Additional supporting information:  crystallographic information; 3D view; checkCIF report

## Figures and Tables

**Table 1:** Hydrogen-bond geometry (Å, °)

*D*—H⋯*A*	*D*—H	H⋯*A*	*D*⋯*A*	*D*—H⋯*A*
O4—H4⋯N3	0.82	1.92	2.637 (3)	146
O2—H2⋯N1	0.82	1.92	2.635 (3)	145
C23—H23⋯N2^i^	0.93	2.57	3.446 (4)	158
C8—H8⋯N4^ii^	0.93	2.60	3.444 (4)	152
C12—H12⋯O1^iii^	0.93	2.46	3.391 (3)	176
C27—H27⋯O3^iii^	0.93	2.52	3.444 (3)	175

Symmetry codes: (i) 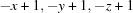; (ii) 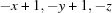; (iii) 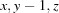.

**Table 2:** Experimental details

Crystal data	
Chemical formula	C_15_H_12_N_2_O_2_
*M*_r_	252.27
Crystal system, space group	Monoclinic, *P*2_1_/*n*
Temperature (K)	293
*a*, *b*, *c* (Å)	14.3173 (11), 13.0633 (9), 14.5450 (11)
β (°)	110.264 (6)
*V* (Å^3^)	2552.0 (3)
*Z*	8
Radiation type	Mo *K*α
μ (mm^−1^)	0.09
Crystal size (mm)	0.77 × 0.51 × 0.28

Data collection	
Diffractometer	Stoe IPDS 2
Absorption correction	Integration (*X-RED32*; Stoe & Cie, 2002 ▸)
*T*_min_, *T*_max_	0.944, 0.981
No. of measured, independent and observed [*I* > 2σ(*I*)] reflections	16144, 4514, 1853
*R*_int_	0.065
(sin θ/λ)_max_ (Å^−1^)	0.596

Refinement	
*R*[*F* ^2^ > 2σ(*F* ^2^)], *wR*(*F* ^2^), *S*	0.043, 0.106, 0.80
No. of reflections	4514
No. of parameters	347
H-atom treatment	H-atom parameters constrained
Δρ_max_, Δρ_min_ (e Å^−3^)	0.10, −0.14

Computer programs: *X-AREA* and *X-RED32* (Stoe & Cie, 2002 ▸), *SHELXT2018* (Sheldrick, 2015*a* ▸), *SHELXL2018* (Sheldrick, 2015*b* ▸), *ORTEP-3 for Windows* and *WinGX* (Farrugia, 2012 ▸), *Mercury* (Macrae *et al.*, 2008 ▸) and *PLATON* (Spek, 2009 ▸).

## supplementary crystallographic information

### Crystal data

**Table:**

C_15_H_12_N_2_O_2_	*F*(000) = 1056
*M**_r_* = 252.27	*D*_x_ = 1.313 Mg m^−^^3^
Monoclinic, *P*2_1_/*n*	Mo *K*α radiation, λ = 0.71073 Å
*a* = 14.3173 (11) Å	Cell parameters from 12734 reflections
*b* = 13.0633 (9) Å	θ = 1.7–30.0°
*c* = 14.5450 (11) Å	µ = 0.09 mm^−^^1^
β = 110.264 (6)°	*T* = 293 K
*V* = 2552.0 (3) Å^3^	Stick, yellow
*Z* = 8	0.77 × 0.51 × 0.28 mm

### Data collection

**Table:**

Stoe IPDS 2 diffractometer	4514 independent reflections
Radiation source: sealed X-ray tube, 12 x 0.4 mm long-fine focus	1853 reflections with *I* > 2σ(*I*)
Plane graphite monochromator	*R*_int_ = 0.065
Detector resolution: 6.67 pixels mm^-1^	θ_max_ = 25.1°, θ_min_ = 2.2°
rotation method scans	*h* = −17→17
Absorption correction: integration (X-RED32; Stoe & Cie, 2002)	*k* = −15→15
*T*_min_ = 0.944, *T*_max_ = 0.981	*l* = −17→17
16144 measured reflections	

### Refinement

**Table:**

Refinement on *F*^2^	0 restraints
Least-squares matrix: full	Hydrogen site location: inferred from neighbouring sites
*R*[*F*^2^ > 2σ(*F*^2^)] = 0.043	H-atom parameters constrained
*wR*(*F*^2^) = 0.106	*w* = 1/[σ^2^(*F*_o_^2^) + (0.0435*P*)^2^] where *P* = (*F*_o_^2^ + 2*F*_c_^2^)/3
*S* = 0.80	(Δ/σ)_max_ < 0.001
4514 reflections	Δρ_max_ = 0.10 e Å^−^^3^
347 parameters	Δρ_min_ = −0.14 e Å^−^^3^

### Special details

**Table:**

Geometry. All esds (except the esd in the dihedral angle between two l.s. planes) are estimated using the full covariance matrix. The cell esds are taken into account individually in the estimation of esds in distances, angles and torsion angles; correlations between esds in cell parameters are only used when they are defined by crystal symmetry. An approximate (isotropic) treatment of cell esds is used for estimating esds involving l.s. planes.

### Fractional atomic coordinates and isotropic or equivalent isotropic displacement parameters (Å^2^)

**Table:**

	*x*	*y*	*z*	*U*_iso_*/*U*_eq_	
O4	0.58870 (18)	0.47768 (14)	0.30079 (13)	0.0819 (6)	
H4	0.593839	0.419694	0.323603	0.123*	
O3	0.63712 (16)	0.77448 (13)	0.58275 (15)	0.0824 (6)	
O1	0.35678 (17)	1.13841 (14)	−0.08120 (17)	0.0898 (7)	
N3	0.61295 (16)	0.33843 (16)	0.43837 (15)	0.0558 (6)	
N1	0.37945 (17)	0.70036 (16)	0.05960 (15)	0.0570 (6)	
O2	0.4072 (2)	0.83829 (15)	0.19874 (14)	0.0917 (7)	
H2	0.409316	0.780745	0.177052	0.138*	
C22	0.61829 (19)	0.51807 (19)	0.47001 (19)	0.0527 (7)	
C24	0.61766 (19)	0.2355 (2)	0.47037 (19)	0.0540 (7)	
C23	0.62435 (19)	0.4118 (2)	0.49956 (19)	0.0569 (7)	
H23	0.636936	0.396297	0.565171	0.068*	
C9	0.37421 (19)	0.59730 (19)	0.02685 (19)	0.0549 (7)	
C8	0.3682 (2)	0.7747 (2)	−0.0008 (2)	0.0589 (7)	
H8	0.354897	0.759991	−0.066688	0.071*	
C14	0.3677 (2)	0.5226 (2)	0.09280 (19)	0.0587 (7)	
C7	0.37551 (19)	0.8803 (2)	0.02964 (19)	0.0553 (7)	
C29	0.6146 (2)	0.1611 (2)	0.39996 (19)	0.0581 (7)	
C17	0.6007 (2)	0.5471 (2)	0.3733 (2)	0.0617 (7)	
C6	0.36283 (19)	0.95656 (19)	−0.0412 (2)	0.0612 (7)	
H6	0.350350	0.937718	−0.105985	0.073*	
N2	0.3453 (2)	0.57423 (19)	0.25515 (18)	0.0911 (9)	
C20	0.6261 (2)	0.6952 (2)	0.5172 (2)	0.0632 (8)	
C10	0.3776 (2)	0.56611 (19)	−0.06333 (19)	0.0635 (8)	
H10	0.381228	0.614757	−0.108635	0.076*	
C21	0.63063 (19)	0.59328 (19)	0.54172 (19)	0.0598 (7)	
H21	0.641935	0.574221	0.606288	0.072*	
C5	0.3684 (2)	1.0581 (2)	−0.0173 (2)	0.0662 (8)	
C25	0.6209 (2)	0.20343 (19)	0.5621 (2)	0.0643 (8)	
H25	0.622114	0.251235	0.609905	0.077*	
C15	0.3572 (2)	0.5527 (2)	0.1837 (2)	0.0652 (8)	
C2	0.3940 (2)	0.9085 (2)	0.1262 (2)	0.0651 (8)	
C11	0.3755 (2)	0.4634 (2)	−0.0860 (2)	0.0708 (8)	
H11	0.376934	0.443640	−0.146878	0.085*	
C13	0.3682 (2)	0.4193 (2)	0.0698 (2)	0.0693 (8)	
H13	0.366432	0.369901	0.115268	0.083*	
C19	0.6079 (2)	0.7230 (2)	0.4211 (2)	0.0749 (9)	
H19	0.603749	0.791933	0.404294	0.090*	
C28	0.6149 (2)	0.0575 (2)	0.4218 (2)	0.0725 (9)	
H28	0.612046	0.008688	0.374315	0.087*	
C26	0.6224 (2)	0.0999 (2)	0.5823 (2)	0.0720 (8)	
H26	0.625365	0.078738	0.644307	0.086*	
C12	0.3712 (2)	0.3899 (2)	−0.0199 (2)	0.0730 (9)	
H12	0.370435	0.320812	−0.035801	0.088*	
C30	0.6120 (3)	0.1939 (2)	0.3053 (2)	0.0780 (10)	
C18	0.5957 (2)	0.6504 (2)	0.3502 (2)	0.0773 (9)	
H18	0.584003	0.670547	0.285793	0.093*	
C27	0.6195 (2)	0.0277 (2)	0.5129 (2)	0.0738 (9)	
H27	0.620696	−0.041524	0.528119	0.089*	
N4	0.6109 (3)	0.2189 (2)	0.2296 (2)	0.1178 (12)	
C3	0.4003 (2)	1.0116 (2)	0.1505 (2)	0.0839 (10)	
H3	0.413366	1.031176	0.215218	0.101*	
C4	0.3873 (2)	1.0848 (2)	0.0794 (2)	0.0790 (9)	
H4A	0.391329	1.153637	0.096722	0.095*	
C16	0.6543 (2)	0.7483 (2)	0.6815 (2)	0.0889 (10)	
H16A	0.599599	0.708154	0.685334	0.133*	
H16B	0.660293	0.809549	0.719536	0.133*	
H16C	0.714693	0.709297	0.706679	0.133*	
C1	0.3355 (3)	1.1138 (2)	−0.1815 (2)	0.0939 (10)	
H1A	0.328552	1.175673	−0.218800	0.141*	
H1B	0.274610	1.075385	−0.205212	0.141*	
H1C	0.388875	1.073549	−0.188179	0.141*	

### Atomic displacement parameters (Å^2^)

**Table:**

	*U*^11^	*U*^22^	*U*^33^	*U*^12^	*U*^13^	*U*^23^
O4	0.1348 (19)	0.0579 (12)	0.0586 (12)	0.0127 (13)	0.0407 (13)	0.0018 (10)
O3	0.1213 (18)	0.0449 (12)	0.0829 (15)	−0.0003 (12)	0.0377 (13)	−0.0080 (12)
O1	0.1321 (19)	0.0433 (12)	0.0982 (17)	−0.0020 (12)	0.0454 (15)	0.0078 (12)
N3	0.0731 (16)	0.0453 (14)	0.0549 (14)	0.0019 (12)	0.0296 (12)	−0.0004 (12)
N1	0.0792 (17)	0.0386 (14)	0.0591 (14)	0.0006 (12)	0.0315 (12)	0.0003 (12)
O2	0.156 (2)	0.0615 (14)	0.0606 (13)	0.0064 (15)	0.0410 (14)	0.0006 (11)
C22	0.0614 (18)	0.0438 (16)	0.0571 (17)	0.0037 (14)	0.0259 (14)	0.0014 (14)
C24	0.063 (2)	0.0453 (18)	0.0580 (18)	0.0012 (14)	0.0272 (15)	−0.0011 (15)
C23	0.074 (2)	0.0490 (18)	0.0517 (16)	−0.0013 (15)	0.0267 (14)	0.0016 (14)
C9	0.0687 (19)	0.0421 (16)	0.0589 (18)	0.0007 (14)	0.0286 (15)	−0.0004 (15)
C8	0.082 (2)	0.0456 (17)	0.0568 (17)	0.0022 (15)	0.0343 (16)	−0.0030 (15)
C14	0.0743 (19)	0.0505 (17)	0.0574 (17)	0.0002 (14)	0.0304 (14)	0.0022 (14)
C7	0.067 (2)	0.0416 (17)	0.0649 (19)	−0.0010 (15)	0.0320 (16)	−0.0052 (15)
C29	0.074 (2)	0.0483 (18)	0.0567 (17)	−0.0024 (14)	0.0286 (15)	−0.0040 (14)
C17	0.078 (2)	0.0522 (18)	0.0600 (18)	0.0069 (15)	0.0306 (15)	0.0017 (15)
C6	0.076 (2)	0.0463 (17)	0.0664 (18)	0.0008 (15)	0.0318 (15)	0.0000 (14)
N2	0.135 (2)	0.0836 (19)	0.0666 (16)	0.0089 (17)	0.0510 (17)	0.0067 (15)
C20	0.073 (2)	0.0480 (19)	0.072 (2)	0.0020 (15)	0.0300 (16)	−0.0031 (16)
C10	0.090 (2)	0.0508 (18)	0.0568 (17)	0.0030 (16)	0.0339 (16)	0.0013 (14)
C21	0.078 (2)	0.0442 (17)	0.0616 (18)	0.0010 (15)	0.0300 (16)	0.0015 (14)
C5	0.081 (2)	0.0401 (18)	0.082 (2)	−0.0014 (15)	0.0343 (18)	0.0036 (16)
C25	0.091 (2)	0.0470 (18)	0.0618 (19)	0.0014 (16)	0.0349 (16)	−0.0023 (14)
C15	0.090 (2)	0.0519 (17)	0.0593 (18)	0.0033 (15)	0.0325 (16)	0.0088 (14)
C2	0.087 (2)	0.0524 (18)	0.0605 (18)	−0.0021 (16)	0.0314 (16)	−0.0030 (16)
C11	0.099 (2)	0.0550 (19)	0.0659 (19)	0.0016 (17)	0.0384 (17)	−0.0044 (16)
C13	0.093 (2)	0.0448 (18)	0.077 (2)	0.0010 (16)	0.0384 (17)	0.0082 (15)
C19	0.103 (3)	0.0473 (18)	0.082 (2)	0.0093 (16)	0.041 (2)	0.0135 (17)
C28	0.094 (2)	0.055 (2)	0.075 (2)	−0.0056 (17)	0.0380 (18)	−0.0113 (17)
C26	0.097 (2)	0.058 (2)	0.0692 (19)	0.0004 (18)	0.0383 (18)	0.0091 (16)
C12	0.097 (2)	0.0472 (19)	0.082 (2)	−0.0035 (17)	0.0399 (19)	−0.0055 (17)
C30	0.120 (3)	0.055 (2)	0.069 (2)	−0.0126 (18)	0.045 (2)	−0.0138 (17)
C18	0.114 (3)	0.059 (2)	0.0673 (19)	0.0143 (18)	0.0419 (19)	0.0152 (17)
C27	0.093 (2)	0.0448 (19)	0.085 (2)	−0.0026 (16)	0.0335 (19)	0.0019 (17)
N4	0.211 (4)	0.083 (2)	0.075 (2)	−0.029 (2)	0.069 (2)	−0.0150 (16)
C3	0.117 (3)	0.060 (2)	0.071 (2)	0.0035 (19)	0.0285 (19)	−0.0132 (18)
C4	0.107 (3)	0.0425 (18)	0.086 (2)	−0.0008 (17)	0.032 (2)	−0.0097 (18)
C16	0.117 (3)	0.070 (2)	0.081 (2)	−0.002 (2)	0.037 (2)	−0.0194 (18)
C1	0.129 (3)	0.070 (2)	0.093 (2)	0.007 (2)	0.052 (2)	0.021 (2)

### Geometric parameters (Å, º)

**Table:**

O4—C17	1.356 (3)	C20—C21	1.374 (3)
O4—H4	0.8200	C20—C19	1.379 (4)
O3—C20	1.380 (3)	C10—C11	1.379 (3)
O3—C16	1.413 (3)	C10—H10	0.9300
O1—C5	1.373 (3)	C21—H21	0.9300
O1—C1	1.420 (3)	C5—C4	1.382 (4)
N3—C23	1.279 (3)	C25—C26	1.383 (3)
N3—C24	1.417 (3)	C25—H25	0.9300
N1—C8	1.282 (3)	C2—C3	1.387 (4)
N1—C9	1.421 (3)	C11—C12	1.375 (4)
O2—C2	1.361 (3)	C11—H11	0.9300
O2—H2	0.8200	C13—C12	1.376 (4)
C22—C17	1.393 (3)	C13—H13	0.9300
C22—C21	1.399 (3)	C19—C18	1.367 (4)
C22—C23	1.447 (3)	C19—H19	0.9300
C24—C25	1.384 (3)	C28—C27	1.361 (4)
C24—C29	1.401 (3)	C28—H28	0.9300
C23—H23	0.9300	C26—C27	1.371 (4)
C9—C10	1.390 (3)	C26—H26	0.9300
C9—C14	1.394 (3)	C12—H12	0.9300
C8—C7	1.442 (3)	C30—N4	1.143 (3)
C8—H8	0.9300	C18—H18	0.9300
C14—C13	1.392 (3)	C27—H27	0.9300
C14—C15	1.436 (4)	C3—C4	1.373 (4)
C7—C2	1.386 (3)	C3—H3	0.9300
C7—C6	1.398 (3)	C4—H4A	0.9300
C29—C28	1.390 (3)	C16—H16A	0.9600
C29—C30	1.430 (4)	C16—H16B	0.9600
C17—C18	1.388 (3)	C16—H16C	0.9600
C6—C5	1.367 (3)	C1—H1A	0.9600
C6—H6	0.9300	C1—H1B	0.9600
N2—C15	1.145 (3)	C1—H1C	0.9600
			
C17—O4—H4	109.5	C26—C25—H25	120.2
C20—O3—C16	117.3 (2)	C24—C25—H25	120.2
C5—O1—C1	117.1 (2)	N2—C15—C14	177.2 (3)
C23—N3—C24	120.2 (2)	O2—C2—C7	122.2 (2)
C8—N1—C9	120.6 (2)	O2—C2—C3	118.5 (3)
C2—O2—H2	109.5	C7—C2—C3	119.3 (3)
C17—C22—C21	119.6 (2)	C12—C11—C10	121.0 (3)
C17—C22—C23	122.1 (2)	C12—C11—H11	119.5
C21—C22—C23	118.3 (2)	C10—C11—H11	119.5
C25—C24—C29	118.5 (2)	C12—C13—C14	120.2 (3)
C25—C24—N3	125.8 (2)	C12—C13—H13	119.9
C29—C24—N3	115.6 (2)	C14—C13—H13	119.9
N3—C23—C22	122.1 (2)	C18—C19—C20	120.8 (3)
N3—C23—H23	118.9	C18—C19—H19	119.6
C22—C23—H23	118.9	C20—C19—H19	119.6
C10—C9—C14	118.5 (2)	C27—C28—C29	119.7 (3)
C10—C9—N1	125.4 (2)	C27—C28—H28	120.1
C14—C9—N1	116.1 (2)	C29—C28—H28	120.1
N1—C8—C7	122.4 (2)	C27—C26—C25	121.5 (3)
N1—C8—H8	118.8	C27—C26—H26	119.3
C7—C8—H8	118.8	C25—C26—H26	119.3
C13—C14—C9	120.4 (2)	C11—C12—C13	119.4 (3)
C13—C14—C15	119.8 (2)	C11—C12—H12	120.3
C9—C14—C15	119.7 (2)	C13—C12—H12	120.3
C2—C7—C6	119.2 (2)	N4—C30—C29	179.0 (4)
C2—C7—C8	122.3 (3)	C19—C18—C17	120.6 (3)
C6—C7—C8	118.6 (2)	C19—C18—H18	119.7
C28—C29—C24	120.8 (3)	C17—C18—H18	119.7
C28—C29—C30	120.6 (3)	C28—C27—C26	120.0 (3)
C24—C29—C30	118.7 (2)	C28—C27—H27	120.0
O4—C17—C18	118.7 (2)	C26—C27—H27	120.0
O4—C17—C22	122.3 (2)	C4—C3—C2	120.3 (3)
C18—C17—C22	119.1 (3)	C4—C3—H3	119.8
C5—C6—C7	121.5 (3)	C2—C3—H3	119.8
C5—C6—H6	119.2	C3—C4—C5	121.2 (3)
C7—C6—H6	119.2	C3—C4—H4A	119.4
C21—C20—C19	119.6 (3)	C5—C4—H4A	119.4
C21—C20—O3	124.4 (3)	O3—C16—H16A	109.5
C19—C20—O3	116.0 (3)	O3—C16—H16B	109.5
C11—C10—C9	120.4 (3)	H16A—C16—H16B	109.5
C11—C10—H10	119.8	O3—C16—H16C	109.5
C9—C10—H10	119.8	H16A—C16—H16C	109.5
C20—C21—C22	120.3 (2)	H16B—C16—H16C	109.5
C20—C21—H21	119.8	O1—C1—H1A	109.5
C22—C21—H21	119.8	O1—C1—H1B	109.5
C6—C5—O1	125.9 (3)	H1A—C1—H1B	109.5
C6—C5—C4	118.5 (3)	O1—C1—H1C	109.5
O1—C5—C4	115.6 (3)	H1A—C1—H1C	109.5
C26—C25—C24	119.6 (3)	H1B—C1—H1C	109.5
			
C23—N3—C24—C25	9.1 (4)	C23—C22—C21—C20	179.6 (3)
C23—N3—C24—C29	−173.6 (2)	C7—C6—C5—O1	−179.8 (3)
C24—N3—C23—C22	−178.8 (2)	C7—C6—C5—C4	0.2 (4)
C17—C22—C23—N3	−0.7 (4)	C1—O1—C5—C6	1.1 (4)
C21—C22—C23—N3	179.3 (3)	C1—O1—C5—C4	−178.9 (3)
C8—N1—C9—C10	13.8 (4)	C29—C24—C25—C26	0.8 (4)
C8—N1—C9—C14	−167.8 (3)	N3—C24—C25—C26	178.1 (3)
C9—N1—C8—C7	−178.4 (2)	C6—C7—C2—O2	−180.0 (3)
C10—C9—C14—C13	2.3 (4)	C8—C7—C2—O2	0.4 (4)
N1—C9—C14—C13	−176.2 (3)	C6—C7—C2—C3	−0.5 (4)
C10—C9—C14—C15	−175.7 (3)	C8—C7—C2—C3	179.9 (3)
N1—C9—C14—C15	5.8 (4)	C9—C10—C11—C12	−0.8 (5)
N1—C8—C7—C2	−0.8 (4)	C9—C14—C13—C12	−2.5 (5)
N1—C8—C7—C6	179.6 (3)	C15—C14—C13—C12	175.5 (3)
C25—C24—C29—C28	0.0 (4)	C21—C20—C19—C18	−1.0 (5)
N3—C24—C29—C28	−177.6 (3)	O3—C20—C19—C18	−180.0 (3)
C25—C24—C29—C30	−179.5 (3)	C24—C29—C28—C27	−0.8 (4)
N3—C24—C29—C30	2.9 (4)	C30—C29—C28—C27	178.7 (3)
C21—C22—C17—O4	179.6 (3)	C24—C25—C26—C27	−0.8 (5)
C23—C22—C17—O4	−0.4 (4)	C10—C11—C12—C13	0.6 (5)
C21—C22—C17—C18	0.0 (4)	C14—C13—C12—C11	1.1 (5)
C23—C22—C17—C18	179.9 (3)	C20—C19—C18—C17	0.6 (5)
C2—C7—C6—C5	0.1 (4)	O4—C17—C18—C19	−179.7 (3)
C8—C7—C6—C5	179.7 (3)	C22—C17—C18—C19	0.0 (5)
C16—O3—C20—C21	0.1 (4)	C29—C28—C27—C26	0.9 (5)
C16—O3—C20—C19	179.0 (3)	C25—C26—C27—C28	−0.1 (5)
C14—C9—C10—C11	−0.7 (4)	O2—C2—C3—C4	−179.9 (3)
N1—C9—C10—C11	177.6 (3)	C7—C2—C3—C4	0.7 (5)
C19—C20—C21—C22	1.0 (4)	C2—C3—C4—C5	−0.4 (5)
O3—C20—C21—C22	179.8 (3)	C6—C5—C4—C3	−0.1 (5)
C17—C22—C21—C20	−0.4 (4)	O1—C5—C4—C3	180.0 (3)

### Hydrogen-bond geometry (Å, º)

**Table:**

*D*—H···*A*	*D*—H	H···*A*	*D*···*A*	*D*—H···*A*
O4—H4···N3	0.82	1.92	2.637 (3)	146
O2—H2···N1	0.82	1.92	2.635 (3)	145
C23—H23···N2^i^	0.93	2.57	3.446 (4)	158
C8—H8···N4^ii^	0.93	2.60	3.444 (4)	152
C12—H12···O1^iii^	0.93	2.46	3.391 (3)	176
C27—H27···O3^iii^	0.93	2.52	3.444 (3)	175

Symmetry codes: (i) −*x*+1, −*y*+1, −*z*+1; (ii) −*x*+1, −*y*+1, −*z*; (iii) *x*, *y*−1, *z*.
